# Treatment of Complete Anal Stricture after Diverting Colostomy for Fournier's Gangrene

**DOI:** 10.1155/2017/2062157

**Published:** 2017-01-31

**Authors:** Kenji Okumura, Tadao Kubota, Kazuhiro Nishida, Alan Kawarai Lefor, Ken Mizokami

**Affiliations:** ^1^Department of Surgery, Tokyo Bay Urayasu-Ichikawa Medical Center (Noguchi Memorial Institution Hospital), Urayasu, Japan; ^2^Department of Surgery, Uwamachi General Hospital, Yokosuka, Japan; ^3^Department of Surgery, Jichi Medical University, Tochigi, Japan

## Abstract

*Background*. Anal stenosis is a rare but serious complication of anorectal surgery. Severe anal stenosis is a challenging condition.* Case Presentation*. A 70-year-old Japanese man presented with a ten-hour history of continuous anal pain due to incarcerated hemorrhoids. He had a history of reducible internal hemorrhoids and was followed for 10 years. He had a fever and nonreducible internal hemorrhoids surrounding necrotic soft tissues. He was diagnosed as Fournier's gangrene and treated with debridement and diverting colostomy. He needed temporary continuous renal replacement therapy and was discharged on postoperative day 39. After four months, severe anal stenosis was found on physical examination, and total colonoscopy showed a complete anal stricture. The patient was brought to the operating room and underwent colostomy closure and anoplasty. He recovered without any complications.* Conclusion*. We present a first patient with a complete anal stricture after diverting colostomy treated with anoplasty and stoma closure. This case reminds us of the assessment of distal bowel conduit and might suggest that anoplasty might be considered in the success of the colostomy closure.

## 1. Background

Anal stenosis is a serious complication of anorectal surgery and can complicate hemorrhoidectomy in up to 5−10% of patients, particularly patients in whom large areas of anoderm and rectal mucosa from the lining of the anal canal are denuded [1, 2]. It can also occur after other anorectal surgical procedures [1–3].

Treatment, both medical and surgical, should be decided based on severity of the stenosis [1–3]. For severe anal stenosis, one should consider surgical interventions including anoplasty [1–4]. Various types of anal stenosis have been reported, but there are no previous reports of a complete anal stricture. We present a patient with a complete anal stricture after diverting colostomy for Fournier's gangrene.

## 2. Case Presentation

**Figure 1:**
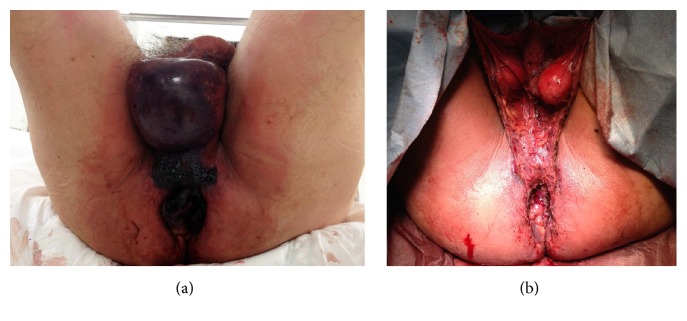
(a) Necrotic incarcerated hemorrhoids and necrotic perineal tissues. (b) Wound after removal of necrotic hemorrhoids and debridement of necrotic tissues.

A 70-year-old Japanese man presented with a ten-hour history of continuous anal pain due to incarcerated hemorrhoids. He had a history of reducible internal hemorrhoids and was followed for 10 years. His weight was 70 kg and BMI 26 kg/m^2^. The remainder of the physical examination revealed a fever of 38.3°C, blood pressure 99/67 mmHg, HR 142/min, and RR 29/min. The skin of the scrotum was dark red and associated with subcutaneous emphysema. Nonreducible internal hemorrhoids were seen (Figure 1(a)). Laboratory results showed white blood cell count 9300/uL (normal range: 3100–8800/uL), neutrophils 77.5% (50–70%), hematocrit 46% (normal range: 40–54%), platelet count 13.9 × 10^4^/uL (15–35 × 10^4^/uL), C-reactive protein 4.04 mg/dL (0–0.29 mg/dL), blood urea nitrogen 21.2 mg/dL (8–22 mg/dL), creatinine 2.86 mg/dL (0.61–1.04 mg/dL), sodium 142 mEq/L (138–146 mEq/L), potassium 3.2 mEq/L (3.6–4.9 mEq/L), pH 7.399 (7.35–7.45), bicarbonate 14.6 mmol/L (22–26 mmol/L), creatinine kinase 6789 U/L (60–287 U/L), and lactate 103 mg/dL (6.3–18.9 mg/dL). Fournier's gangrene severity index score was 13. Computed tomography scan of the pelvis showed edema of the perianal soft tissues. The patient was diagnosed with septic shock due to a necrotizing soft tissue infection secondary to incarcerated hemorrhoids. After fluid resuscitation and antibiotic administration, emergent debridement of necrotic tissues and hemorrhoidectomy were performed (Figure 1(b)). Additional debridement and a diverting loop colostomy were performed two days later. Tissue cultures showed Klebsiella pneumonia and Group G streptococcus. Renal replacement therapy was used to treat acute kidney injury, but he gradually improved, and his kidney function normalized. He was discharged on postoperative day 39.

**Figure 2:**
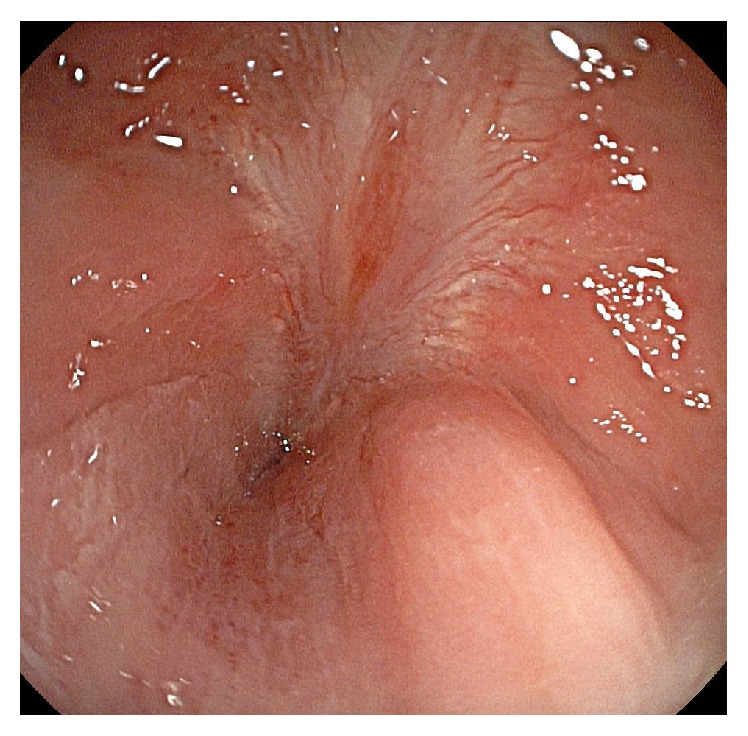
Colonoscopy: total stenosis with scarring.

After four months, we planned to perform a colostomy closure. Severe anal stenosis was found on physical examination, and total colonoscopy was performed before surgery, which showed a complete anal stricture (Figure 2). The patient was brought to the operating room and underwent colostomy closure and anoplasty (V-Y flap). During followup, anal stenosis was seen on rectal examination, but the patient did not have significant symptoms. One year later, he appeared fully recovered with no complications.

## 3. Discussion

Anal stricture is a tight narrowing of the anal canal that may interfere with defecation [2, 4]. The most common cause of an anal stricture is iatrogenic, from excision of anoderm during surgery for hemorrhoids, anal warts, or fistulae-in-ano [2, 4]. Anal strictures are usually diagnosed based on a history of difficult or painful bowel movements. Suspicion of anal stenosis is heightened by a history of hemorrhoidectomy, Crohn's disease, or laxative abuse [2, 4]. The diagnosis of anal stenosis is usually made based on a complete history and physical examination. It is difficult to diagnose anal stenosis in patients who have a diverting stoma because they do not pass stool through the anus and thus have no anal symptoms.

Fournier's gangrene has been reported after treatment of hemorrhoids but complete anal stricture after the treatment of Fournier's gangrene has not previously been reported. Aggressive and repeated debridement is the most crucial step in the treatment of a patient with Fournier's gangrene [5]. Although a diverting stoma can help promote wound healing by avoiding fecal contamination, it should be performed only in selected cases, such as Fournier's gangrene involving the anorectal area and sphincter, because stomas are associated with increased morbidity [5].

In the present patient, a complete anal stricture developed in association with debridement and diverting colostomy. Stoma closure was planned, and during preoperative workup, a complete anal stricture was found. Therefore, it was considered necessary to perform stoma closure and anoplasty at the same time.

To prevent anal stenosis, surgeons should avoid excessive excision of normal anoderm. However, in patients with Fournier's gangrene this may be difficult to avoid as wide debridement is essential. After anorectal procedures, a meticulous rectal examination is essential to diagnose anal stenosis early, especially in high-risk patients. Additionally, malignancy should be ruled out in the remaining bowel prior to stoma closure [5].

In summary, this is the first report of a complete anal stricture after treatment of Fournier's gangrene with debridement and a diverting colostomy. This case reminds us of the assessment of distal bowel conduit and suggests that anoplasty might be considered in patients with severe anal stenosis undergoing stoma closure.

## Additional Points

*Institutional Review Board Statement*. This report is a case report and does not need a review of IRB.

*Biostatistics Statement.* There is no statistical analysis.

## Data Access

No additional data are available.
